# A Rare Manifestation of Discoid Lupus Erythematosus Solely in the Lower Eyelid of a Young Man

**DOI:** 10.7759/cureus.47002

**Published:** 2023-10-13

**Authors:** Konstantina Bachtalia, Konstantina Frangia-Tsivou, Andreas Patelis, Sotiria Palioura

**Affiliations:** 1 Biomedical Sciences, University of West Attica, Athens, GRC; 2 Pathology, HistoBio Diagnosis Pathology Lab, Athens, GRC; 3 Ophthalmology, Ophthalmology Clinic, Athens, GRC; 4 Ophthalmology, Bascom Palmer Eye Institute, Miami, USA

**Keywords:** eyelid edema, eyelid erythema, ocular discoid lesions, chronic blepharitis, sebaceous cell carcinoma, eyelid discoid lupus

## Abstract

Discoid lupus erythematosus (DLE)-associated edema and erythema on the lower eyelid as the only manifestation of the disease is a rare clinical entity. Persistent discoid lupus-related lower eyelid manifestations are challenging to diagnose, can be mistaken for blepharitis or malignancies, and often require histopathological evaluation. If left untreated, the condition can progress, among others, to conjunctival scarring or symblepharon formation. Thus, early identification and management of the disease entity is of the utmost significance. We present a rare case of a young patient with enduring, unilateral lower eyelid edema and erythema that had been managed as blepharitis for several years. No other related cutaneous involvement was detected on the eyelids, face, or body. Following a lower eyelid biopsy and histopathological assessment, the patient was diagnosed with underlying DLE. This case report reviews the previous literature, discusses a differentiation strategy from other relevant pathologies, such as blepharitis and sebaceous cell carcinoma, and highlights the implemented diagnostic procedures.

## Introduction

Discoid lupus erythematosus (DLE) is a unique subtype of chronic cutaneous lupus erythematosus (CLE) [1]. This immune-mediated variant primarily affects UV-exposed areas such as the head, neck, and face [2]. It typically presents with localized, erythematous scaly lesions, gradually progressing to atrophic scarring along with pigmentary alterations [1,3].

The ocular manifestations of DLE comprise distinctive erythematous discoid plaques on the eyelids, pigmentary changes of the periocular skin, madarosis, trichiasis, lid margin telangiectasias, disruption of the eyelid margin, conjunctival scarring, entropion, ectropion, and symblepharon formation [2,3]. Such persistent clinical signs may raise the suspicion for other malignant conditions, including squamous or sebaceous cell carcinoma [4,5].

Discoid lupus-associated erythematous lesions on the eyelids are rare and detected in barely 6% of DLE cases [1,5]. Cases of eyelid involvement with well-characterized erythematous lesions on the periorbita or near the outer eyelid margins have been previously described [1,6-9]. In the reported DLE cases with eyelid abnormalities, patients already carry a prior diagnosis of DLE based on the presence of concomitant peripheral, predominantly facial, cutaneous lesions [3,4,6]. Moreover, the incidence of the exclusive manifestation of DLE on the main palpebral area of a single lower eyelid, which can be misdiagnosed as a different pathology such as blepharitis, is exceptionally scarce and inadequately reported in the literature.

Here, we report a case with unusual persistent unilateral lower eyelid edema and erythema as the only manifestation of DLE. The patient had been misdiagnosed with chronic blepharitis for several years before presentation by multiple providers. A summary of the previous literature on DLE lower eyelid involvement is also provided and illustrates its scarcity. Finally, a differential diagnosis from other relevant pathologies is discussed and the implemented therapeutic procedures are highlighted.

## Case presentation

A 24-year-old otherwise healthy Caucasian male presented with a three-year history of persistent unilateral lower eyelid edema and erythema. The patient had been previously managed with eyelid hygiene, a combination of topical antibiotic and steroid regimens, and tacrolimus 0.03% ointment for presumed enduring blepharitis and meibomian gland dysfunction without much relief. Meibomian gland biopsy had been performed two years before presentation indicating no atypia or dysplasia of the squamous epithelium. However, persistent clinical signs and patient-reported exacerbation of ocular symptoms prompted additional diagnostic considerations and exclusion of any related underlying pathology.

**Figure 1:**
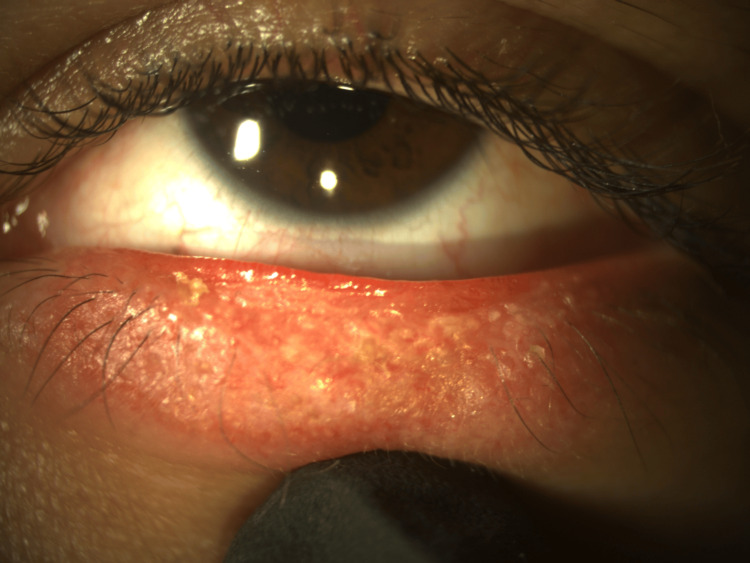
External photograph of the patient’s left lower eyelid. Extended central erythematous lesion with peripheral hyperpigmentation, distinctive central depigmentation, madarosis, and adherent scaling and scarring.

External examination revealed an extended central erythematous lesion on the left lower eyelid with peripheral hyperpigmentation, distinctive central depigmentation along with madarosis, adherent scaling, and scarring (Figure 1).

**Figure 2:**
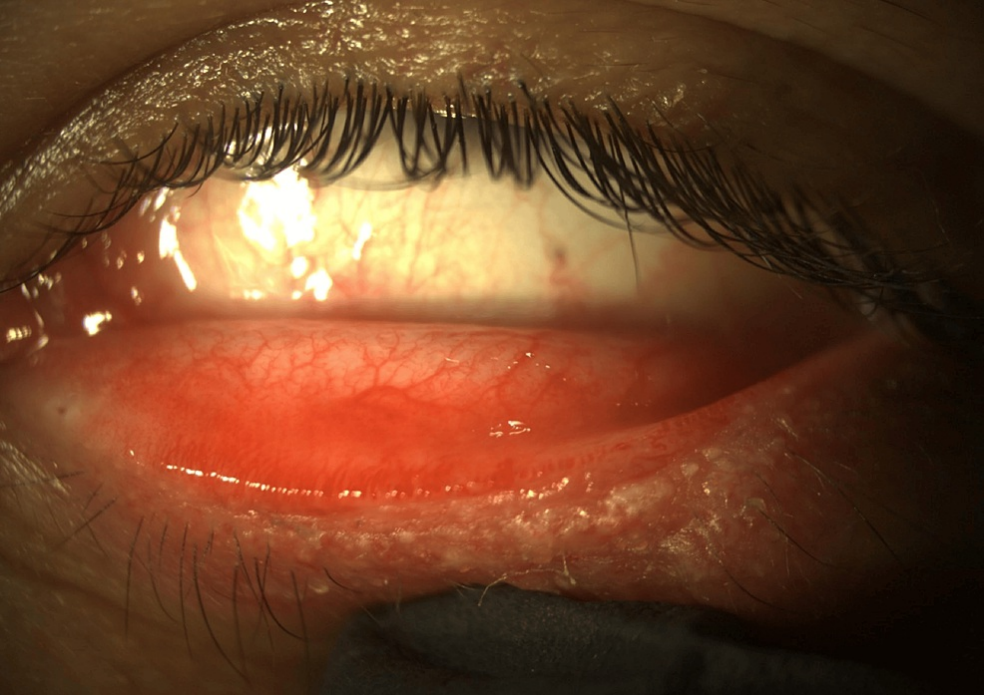
External photograph of the patient’s left lower eyelid. Prominent thickening and inflammation across the eyelid margin, derangement of the mucocutaneous junction, hypertrophy of the meibomian glands, and focal conjunctival injection.

No associated cutaneous findings on the scalp, face, or body of the patient were detected. Slit-lamp bio-microscopy showed prominent thickening and inflammation across the left lower eyelid margin, which was associated with derangement of the mucocutaneous junction, hypertrophy of the meibomian glands, and focal conjunctival injection (Figure 2). No corneal involvement was identified.

**Figure 3:**
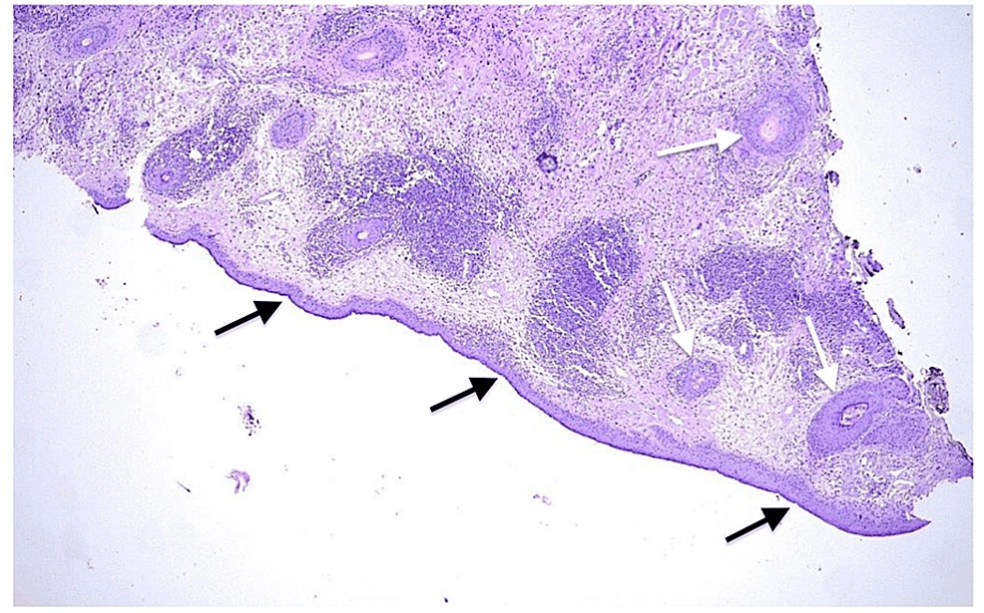
Low-power (×10) magnification of the lower lid biopsy specimen. Typical discoid lupus erythematosus-associated epidermal alterations including acanthosis (black arrow) and follicular plugging (white arrow) are present.

**Figure 4:**
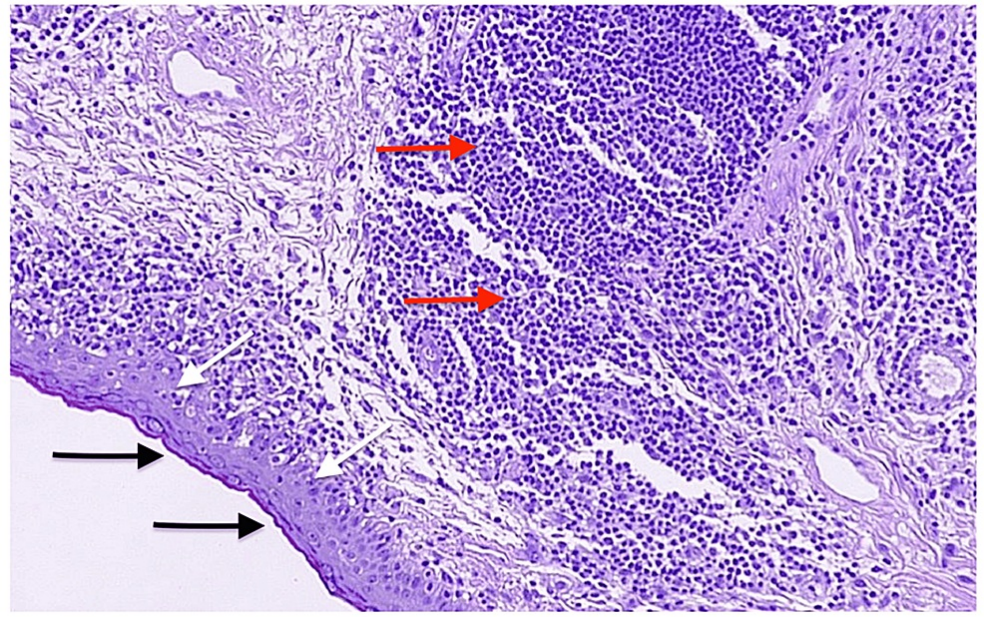
Low-power (×10) magnification of the lower lid biopsy specimen. Typical discoid lupus erythematosus-associated epidermal alterations including acanthosis (black arrow), epidermal atrophy (white arrow), and a lymphocytic infiltrate (red arrow) are present.

A biopsy of the lower eyelid skin was performed under monitored anesthesia care and controlled intraoperative hemostasis. Histopathologic examination revealed mild hyperkeratosis and areas of mild-to-moderate acanthosis alternating with epidermal atrophy and follicular plugging (Figures 3, 4).

**Figure 5:**
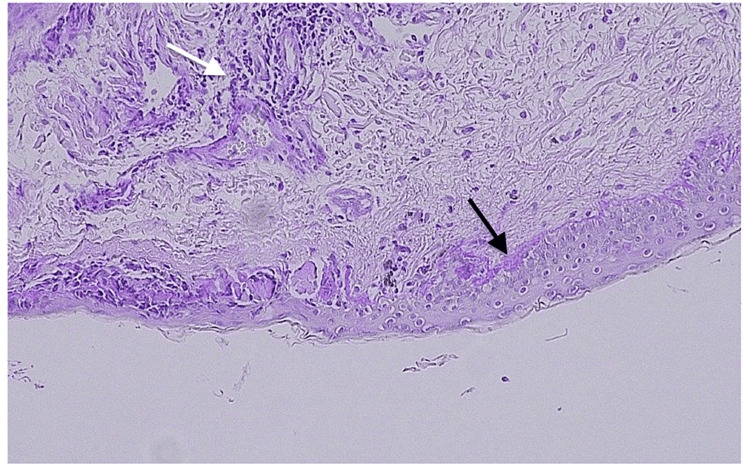
Periodic acid–Schiff staining of the lower lid biopsy specimen. Discoid lupus erythematosus-consistent perivascular inflammatory lymphocytic infiltrates (white arrow) and basement membrane zone thickening (black arrow) are present.

**Figure 6:**
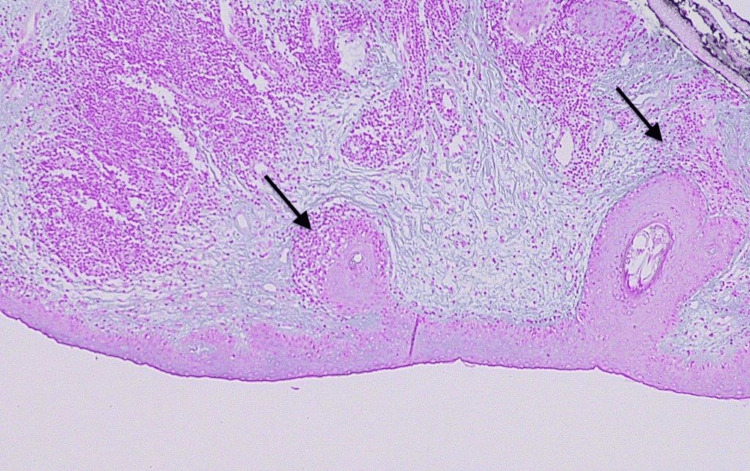
Periodic acid–Schiff staining of the lower lid biopsy specimen. Discoid lupus erythematosus-consistent periadnexal inflammatory lymphocytic infiltrates (black arrow) are present.

Vacuolar basal cell degeneration, albeit mild, was also featured in the specimen. Periodic acid-Schiff staining revealed mild thickening of the basement membrane zone. Moderate-to-severe chronic inflammatory lymphocytic infiltrates were observed in a perivascular and periadnexal pattern (Figures 5, 6). Occasional interstitial plasma cell and mast cell infiltrations were also detected. These microscopic findings were consistent with DLE.

The patient was referred to Rheumatology for further workup and management. Serology testing for the presence of autoimmune dysregulation was performed and was negative for the lupus extractable nuclear antigen (ENA) panel (i.e., Ro, La, Sm, RNP, Scl-70, and Jo1). The patient was started on daily hydroxychloroquinolone with resolution of his signs and symptoms at his 18-month follow-up.

## Discussion

DLE can be subdivided into generalized, childhood, and localized DLE [2]. Localized DLE frequently affects areas above the neck, manifesting with skin lesions on the head and face [2]. The ocular manifestations of the localized form include unilateral swelling and erythema of the upper and lower eyelids (44%) or bilateral upper eyelid involvement (33%) [1].

The incidence of lower eyelid involvement due to underlying DLE is rare with only 28 cases reported in the literature (Table 1) [1-9] Most patients (86%) were females with an average age of 45.3 years (range 19 to 89 years). The reported lower eyelid findings were predominantly treated as blepharitis in up to 43% of the cases, with one case being managed as both blepharitis and allergic dermatitis, one case as both rosacea and allergic dermatitis, and one case as an atypical chalazion. Among the reported cases, only four patients had a diagnosis of DLE before referral to ophthalmology. At presentation, the palpebral abnormalities comprised scaly lesions in 11 (39%) cases with concomitant blepharitis in two cases. Meibomian gland dysfunction but no scaly lesions was identified in three patients. Lower eyelid findings also encompassed marginal irregularities in five (18%) cases, madarosis in 12 (43%) cases, trichiasis in one case, and telangiectasias in three (11%) cases. Conjunctival hyperemia was present in four (14%) of the reported cases. The duration between symptom onset and diagnosis of DLE ranged between two months and 25 years. DLE-associated peripheral cutaneous manifestations were disclosed in 54% of patients with both bilateral and unilateral lower eyelid involvement. Only four (14%) patients, including, similar to our case, two young men, had solely unilateral lower eyelid involvement with no peripheral cutaneous lesions related to DLE at the time of presentation.

**Table 1:** DLE-associated lower eyelid manifestations and management in the reviewed literature. DLE = discoid lupus erythematosus; y/o = years old

Reports	Patient age and gender	Symptom onset up to diagnosis of DLE	Treatment before diagnosis of DLE	Lower eyelid manifestations of DLE	Peripheral cutaneous manifestations of DLE	Treatment after diagnosis of DLE systemic, topical	Outcome
Theisen et al. (2022) [1]	19 y/o male	24 months	Not reported	Unilateral lower eyelid involvement	No peripheral lesions at presentation	Hydroxychloroquine, topical tacrolimus, mometasone	Repeated flares of DLE
	58 y/o female	7 years	Not reported	Bilateral lower eyelid involvement	DLE lesions on the neck and cheeks	Hydroxychloroquine, topical tacrolimus	A flare of DLE at 21 months
	71 y/o female	3 years	Not reported	Bilateral lower eyelid involvement	Cutaneous lesions on both upper eyelids, cheeks, nose, and temples	Hydroxychloroquine, lenalidomide, prednisone, topical tacrolimus, desonide, tofacitinib	A flare of DLE at 7 months
Wang et al. (2021) [7]	28 y/o female	3 years	Not reported	Unilateral scaly plaque, superficial atrophic scars, mild swelling	Well-defined periocular scaly lesions	Hydroxychloroquine, topical tacrolimus	Improvement of lesions on the upper eyelid. Worsening of lesions on the lower eyelid
Jisha et al. (2017) [3]	42 y/o female	1 year	Topical steroids, eyelid hygiene	Unilateral erythematous lesion with minimal scaling	Erythematous patches with scaling on the lower lip, upper chest, ear, and scalp	Hydroxychloroquine, topical steroids	Regression of lesions
	52 y/o female	6 months (relapse of DLE after 4 years)	Topical steroids	Unilateral erythematous lesion with greasy scaling and madarosis	Erythematous patch on the face, near the medial canthus	Hydroxychloroquine, topical steroids	Improvement of symptoms
	37 y/o female	Diagnosis of DLE before referral	-	Unilateral erythematous lesion with scaling, madarosis, and destruction of the eyelid margin	Discoid lesions, close to the inner canthus and over the chest and face	Hydroxychloroquine, topical steroids	Regression of lesions
Galeone et al. (2014) [8]	33 y/o female	3 years	Topical antibiotics and steroids	Unilateral blepharitis, meibomitis, and mild edema	Erythematous scaly patches in proximity to the inner and outer thirds of the eyelid margin	Hydroxychloroquine	Improvement after six weeks, complete resolution of the lesions after 11 weeks
Kopsachilis et al. (2013) [2]	45 y/o female	21 years	Antibiotics, topical steroids, and eyelid hygiene	Bilateral extended madarosis, eyelid margin thickening, severe erythema, and meibomian gland dysfunction	Discoid facial lesions on the chin and nose	Hydroxychloroquine	Reduced inflammation at 2 months, presence of cicatricial ectropion at 6 months
Ghauri et al. (2012) [9]	49 y/o female	10 years	Eyelid hygiene, topical lubricants, and steroids	Unilateral erythema, central erythematous plaque	Not reported	Hydroxychloroquine, topical steroids	Considerable improvement at 3 months
	47 y/o female	11 years	Eyelid hygiene, topical and oral antibiotics	Unilateral irritation, scarring, scaly plaque, telangiectasia, and madarosis	Erythematous annular plaque on the left arm	Hydroxychloroquine, topical steroids	Improvement of symptoms at 4 months
	49 y/o female	19 months	Eyelid hygiene, topical antibiotics, and steroids	Unilateral scaly plaque, madarosis, telangiectasia, and conjunctival injection	Not reported	Hydroxychloroquine, intralesional steroid injections, topical tacrolimus	Remission at 1 year
	29 y/o female	2 months	Eyelid hygiene, topical antibiotics, and steroids	Unilateral erythematous scaly plaque, blepharitis telangiectasia	Scaly, red lesion on the left cheek and temporal area	Hydroxychloroquine	Asymptomatic, no recurrence at 1 year
Gupta et al. (2012) [5]	48 y/o female	10 months	Treated as blepharitis	Bilateral depigmentation, slight hypertrophy	No peripheral lesions at presentation	Hydroxychloroquine	Significant resolution of symptoms
	43 y/o female	57 months	Not reported	Unilateral erythematous scaly plaque, eyelid margin ulceration, madarosis, conjunctival hyperemia	No peripheral lesions at presentation	Hydroxychloroquine, oral and intralesional steroids	Significant improvement
	23 y/o male	86 months	Surgically treated as atypical chalazion	Unilateral swelling, erosion, and erythematous nodules	No peripheral lesions at presentation	Hydroxychloroquine	Poor compliance. Worsening of the lesion
	71 y/o female	18 months	Treated as blepharitis and allergic dermatitis	Unilateral irregular lid margin thickening, madarosis	No peripheral lesions at presentation	Hydroxychloroquine	Improvement
Papalas et al. (2011) [4]	33 y/o female	Diagnosis of DLE 3 years before referral	Not reported	Unilateral lesion increasing in size, exposure-type symptoms	Peripheral DLE manifestation on the scalp	CO_2_ laser	Exposure keratopathy
	40 y/o female	6 months	Not reported	Unilateral erythematous scaling lesion, lid margin irregularities, madarosis	Not reported	Maxitrol ointment	Good response
	53 y/o female	60 months	Not reported	Bilateral lower eyelid involvement	Erythematous, brown-crusted papules under the eyelids	Topical antibiotics	Initial response, recurrence of symptoms
	89 y/o male	Several months	Not reported	Unilateral lower eyelid involvement	Enlarging erythematous patch on the cheek	Prednicarbate emollient cream (0.1%)	Persistent
	40 y/o female	24 months	Not reported	Unilateral slowly enlarging lesion	Not reported	Topical tobradex	Good response
	55 y/o female	30 months	Not reported	Unilateral plaque, underlying edema, madarosis	Not reported	Topical tobradex and protopic	Good response
Acharya et al. (2005) [6]	33 y/o male	Diagnosis of DLE before referral	-	Unilateral thickening, erythema, scaling, mild conjunctival injection, meibomitis	Facial lesion	Hydroxychloroquine	Improvement within 6 weeks, stabilization of symptoms at 6 months
	58 y/o female	15 years	Treated as rosacea and as an allergy	Bilateral erythema, scaling, blepharitis, madarosis, and meibomian gland dysfunction	Hypertrophic periocular lesion	Hydroxychloroquine	Improvement within 6 months, no recurrence at 1 year after discontinuation
	29 y/o female	1.5 years	Antibiotics, topical steroids	Unilateral erythema, meibomian gland dysfunction, madarosis	Lupus dermatitis behind the ear and on the scalp	Hydroxychloroquine	Improvement after 4 days, discontinuation after 12 days due to a drug eruption
	54 y/o female	25 years	Doxycycline	Bilateral thickening, erythema, madarosis	Upper lip scarring	Hydroxychloroquine	Improvement in 2 months
	41 y/o female	Diagnosis of DLE before referral	Previously treated for blepharitis	Bilateral, thickening, erythema, meibomian gland dysfunction, chalazia, conjunctival injection, and trichiasis	Skin lesion on the forehead	Hydroxychloroquine	Improvement within 2 weeks

Following DLE diagnosis, the therapeutic management included hydroxychloroquine per os in most cases (79%), in addition to topical corticosteroids, intralesional steroid injections, or topical tacrolimus. Improvement and/or resolution of the eyelid lesions was noted in 82% of the cases. One patient developed cicatricial ectropion due to poor compliance. Treatment solely with topical regimens was reported in 21% of the cases. Patients were treated with topical steroids in combination with antibiotics in three cases and with tacrolimus in one case. One patient was treated with antibiotic ointment alone. However, similar to our case, the persistence of lesions was documented in 40% of these cases, highlighting that a systemic treatment approach is crucial. Recurrence of signs and symptoms was reported in three (11%) cases, indicating that DLE with eyelid involvement can be a chronic and relapsing process.

The pathogenesis of DLE is currently unknown. The prevalence of DLE is thought to be influenced by ethnicity, female gender, and aging [1]. This report represents the fifth case reported in the literature of DLE presenting as long-standing unilateral lower eyelid involvement. Such an uncommon incidence, along with the absence of any alarming DLE peripheral cutaneous anomalies or systemic involvement, has been challenging in terms of diagnosis, prognosis, and management. The differential diagnosis of lower eyelid DLE includes pathologies such as blepharitis, contact dermatitis, rosacea, sebaceous, and squamous cell carcinoma [6,7,9]. Rosacea blepharokeratoconjunctivitis is an inflammatory skin condition that accounts for up to 17% of all cases of periorbital dermatitis and may simulate discoid lupus [4]. However, ocular rosacea predominantly affects both eyelids, showing a more diffuse erythematous pattern. In addition, most rosacea patients present with a history of persistent extraocular erythema, papules, or pustules that precede the palpebral involvement [4].

In lower eyelid DLE, the long-standing refractory swelling and erythema of the eyelid prompts the exclusion of sebaceous gland carcinoma [5]. Although rare, sebaceous gland carcinoma is an exceptionally malignant and aggressive cutaneous tumor. Early identification and appropriate management are of extreme importance to save the patient’s eye and life. Ocular sebaceous carcinoma can imitate numerous benign conditions, including chalazion or posterior blepharitis, and it is strongly associated with madarosis and unilateral blepharoconjunctivitis [10]. In the setting of enduring lower eyelid edema and erythema, differentiation between sebaceous carcinoma and an immune-based disease such as discoid lupus is crucial in terms of prognosis and treatment. Hence, in our case, the suspicion of malignancy dictated the need for biopsy and subsequent histopathological assessment.

Previous studies have reported the histopathological examination criteria for cutaneous lupus, as detected in dermal tissue from extraocular areas. Distinctive features include follicular plugging, vacuolar interface alteration, compact orthokeratosis, and perifollicular inflammation [2,6,9]. In terms of DLE diagnosis, serology testing for ENA or antinuclear antibody (ANA) is most frequently performed. In our case, blood testing revealed a negative lupus ENA panel. This is not surprising as patients with discoid lupus have a lower ANA positivity compared to patients with other subtypes of cutaneous lupus. Positive ANA has been identified in only 22.9% of all DLE cases with ocular involvement [7]. Thus, DLE diagnosis based solely on serological findings is insufficient.

Following accurate DLE diagnosis, the patient was treated with oral hydroxychloroquinolone which is considered the standard of care in the management of DLE. The most frequently prescribed dosage of hydroxychloroquine is 200 mg twice daily, while the mean reported duration of treatment ranges from two weeks to several months [2,6,9]. Proper therapeutic intervention and patient compliance resulted in evident resolution of all signs and symptoms at the patient’s 18-month follow-up visit.

## Conclusions

Persistent unilateral lower eyelid sole manifestations due to DLE are scarce and can be challenging in terms of diagnosis and proper management. Progression of this condition can lead to conjunctival scarring or symblepharon formation. Prompt identification and differentiation from other eye and life-threatening pathologies via clinical and histopathological evaluation are of the utmost importance.
